# Self-Expansion Activities with a Partner as One Path to Well-Being

**DOI:** 10.3390/bs16050700

**Published:** 2026-05-04

**Authors:** Arthur Aron, Jennifer M. Tomlinson

**Affiliations:** 1 Department of Psychology, Stony Brook University, Stony Brook, NY 11794, USA; 2 Department of Psychological and Brain Sciences, Colgate University, Hamilton, NY 13346, USA

**Keywords:** self-expansion, close relationships, shared activities, well-being, relationship satisfaction

## Abstract

The self-expansion model has generated hundreds of studies on the benefits of relationships for the self, but few have explicitly made the link from relationship quality to well-being. In this article we focus on how doing self-expanding activities with a romantic partner can enhance relationship quality and, in turn, enhance well-being. First, we review the basic model; then, we present a section on the overall well-being benefits of strengthening relationships; then, we go through the various areas of study of self-expansion for couples highlighting for each the studies that show not just greater marital satisfaction, but the effect on various specific well-being effects. After one develops a relationship, one becomes accustomed to this self-expansion through including the partner into the self. As a result, the excitement of this inclusion slows over time, and thus relationships can become dull. But we argue that a major way to reinvigorate relationships is by doing novel and challenging (self-expanding) activities together with one’s partner, so that the self-expansion becomes associated with the relationship. We then briefly review many studies (experiments, daily diary, and others) supporting this principle: shared self-expanding activities increase relationship quality. Finally, we briefly discuss research showing that enhancing relationship quality increases well-being. This emphasis on well-being is important because it can help researchers understand the mechanisms behind how a specific form of improving relationships contributes to well-being.

## 1. Introduction

Engaging in new and exciting activities can have benefits for both relationships and the self-concept, and thereby for overall well-being. In this paper we consider how self-expanding activities done with one’s partner can positively affect the relationship, and in this way strengthen well-being and growth. Although the overall self-expansion model has been applied importantly to contexts other than just existing romantic relationships, such as to individual expansion, attraction and forming new relationships, in this review we keep the focus on its major work on existing and long-term romantic relationships. Also, in this review we focus on the findings that explicitly enhance well-being, happiness, health, and personality traits such as self-esteem and secure attachment (including the extent to which they serve as mediators of self-expansion effects or self-expansion mediates the effects on these outcomes). This is to our knowledge the first overall review of the self-expansion literature that explicitly focuses on well-being.

First, we review the basic self-expansion model; then, we present a section on the overall well-being benefits of strengthening relationships; then, we go through many of the various areas of study of self-expansion for couples highlighting for each the studies that show not just greater marital satisfaction, but the effect on various specific well-being effects. We hope this focus will be useful in encouraging future researchers to measure well-being outcomes along with relationship satisfaction. And ultimately, the goal is to be able to improve well-being, which would have important clinical outcomes and implications to share with the public.

## 2. The Basic Self-Expansion Model

The self-expansion model (originating in Aron & Aron, 1986; and tested and refined in many studies) proposes that we have evolved with two primary motivations: survival and self-expansion (growth, increasing knowledge and abilities, status, and so forth). For recent reviews, see Aron et al. (2022) and Emery et al. (2025). The model then proposes that a major way people experience self-expansion is through close relationships in which the other is, to some extent, “included in the self,” so that one feels they have access to the other’s knowledge, resources, and so forth as part of oneself. Inclusion of others in the self has been tested and confirmed in a variety of methods and it has also led to a measure involving overlapping circles (Aron et al., 1991) that has been cited in thousands of studies (for a review focusing specifically on this aspect, see Branand et al., 2019). The process of forming a close relationship is particularly exciting because the inclusion usually happens quickly through a process of rapid self-expansion (which makes forming close relationships exciting and meaningful). However, over time, one gets used to this inclusion and the excitement slows down. Thus, one way people can make long-term relationships feel more exciting and meaningful (and thus making their relationship become more beneficial to the self) was proposed. The primary method proposed (and studied in the self-expansion model) is to regularly engage in exciting and novel activities with the other, so the relationship becomes associated with that sense of rapid self-expansion. In this way, the relationship remains more meaningful and enjoyable and, in turn, promotes greater well-being in a variety of ways.

In terms of the importance of these predictions about effects on relationships, much research has been carried out focusing on how relationships are a very important source of growth. This growth can occur through the process of including others in the self and through engaging in exciting activities (Aron et al., 2022). Feeney and Collins (2015) make a convincing case that relationships promote thriving through support in times of adversity, but also in times of opportunity. If partners are able to maximize the benefits of exciting and novel activities, this can benefit relationships, the self-concept, and, in turn, improve overall well-being (as discussed in some detail shortly).

As the two-dimensional model of self-change points out, self-expansion itself involves the addition of positive self-content to the self-concept and is associated with increased love and satisfaction (Mattingly et al., 2014; McIntyre et al., 2015). Indeed, work on communication and self-expansion finds that the majority of changes to the self through self-expansion are perceived as positive, while around 20% of growth to the self was simultaneously positive and negative, as reported in semi-structured interviews (Dun, 2008). Thus, when a relationship ends, if it is self-expanding, it results in a loss of self, but if the relationship is self-contracting (resulting in loss of positive self-content) or self-adulterating (resulting in added negative self-content), growth can occur (Lewandowski & Bizzoco, 2007; Mattingly et al., 2014).

## 3. Well-Being Benefits of Relational Self-Expansion

For the purposes of this review, we define well-being broadly as increased positive affect, life satisfaction, and anything that can positively contribute to mental or physical health. This is in line with definitions of subjective well-being or hedonic well-being (Diener et al., 1999). There is considerable research regarding the overall relationship quality effects of well-being. Studies have consistently shown relationship quality as a very major predictor of well-being. For example, in a widely cited meta-analysis (Proulx et al., 2007) of 93 studies, relationship quality was positively correlated with well-being, both at individual time points and over time. Further, Dush and Amato (2005) found that married individuals who rated their marital happiness one standard deviation above the mean reported substantially greater subjective well-being, compared to those scoring a standard deviation below the mean. And many studies since have continued to show these effects. And not just overall, but for various aspects of well-being. For example, a classic foundational study (leading to many other studies showing the same effects) found a strong correlation between relationship quality and happiness (Diener & Seligman, 2002).

Similarly, it is well established that relationship quality is a very strong predictor of health and longevity. A classic meta-analysis (Holt-Lunstad et al., 2017) found that relationship quality was a stronger predictor of longevity than even smoking or obesity. And many studies have found positive associations between marital quality and health (Kiecolt-Glaser & Wilson, 2017; Umberson & Karas Montez, 2010). In another large sample, tested six times over a 20-year period, marital quality predicted self-reported health at the next time point, controlling for the previous one, but the reverse direction was not found, supporting the notion of marital quality as a cause of health (Proulx & Snyder-Rivas, 2013). And a very recent study by Shrout et al. (in press), of 373 newlywed couples followed for 16 years (tested at years 1, 3, 7, and 16), found that various measures of marital quality (e.g., spousal responsiveness) predicted overall self-reported health each year.

Relationship quality has also been found to enhance self-esteem and has implications for self-efficacy (e.g., Robinson & Cameron, 2012). In addition, good adult relationships can help offset attachment insecurity through behaviors that help to improve working models of self and others (e.g., Arriaga et al., 2014; Cortes et al., 2020; Jakubiak et al., 2023). Further, high quality relationships can provide a source of strength in times of stress and a relational catalyst in times of opportunity (Feeney & Collins, 2015). Through support, particularly during good times, relationship partners can promote positive affect, gratitude, and help solidify memories (Gable et al., 2004; Gray et al., 2024). Such positive relational experiences build a foundation of emotional capital that partners can draw from during more challenging times (Feeney & Lemay, 2012).

In the following sections we specifically emphasize studies that in addition to relationship benefits show effects (direct or indirect) on well-being.

## 4. Research on Shared Leisure Activities

In a classic study that examined the effect of shared activities, Orthner (1975) argued that the marital period is a critical determinant of the strength of association with marital satisfaction. Although the overall effect for joint activities was positive, he divided the sample into five separate groups based on length of marriage (period 1 was 0–5 years married, II was 6–11 years, III was 12–17 years, etc.) to account for variations based on length of marriage. Orthner found the strongest association for marital periods I and IV (0–5 years and 12–17 years) and almost no association for periods II and III (the 6–11 years and 12–17 years). Orthner reasoned that periods I and IV are critical periods in a marriage where, first, the couple is establishing their marriage and later during period IV, children may be leaving home and the marital relationship must be reestablished. However, Holman and Jacquart (1988) failed to replicate these findings and instead found a totally different pattern of association. Holman and Jacquart argued that Orthner’s 6-year marital periods were arbitrary and that the exact transitions in a marriage might matter more than a cutoff. Thus, there is a debate about the influence of relationship length on the benefit to shared activities. Leisure activities in general seem to enhance affective well-being and cognitive performance for older adults (Luo et al., 2026). In particular, shared leisure activities done with one’s partner are associated with satisfaction (Dobson & Ogolsky, 2022). However, time spent together only predicts life satisfaction in high-quality relationships (Hudson et al., 2020). More importantly, the research emphasized in this paper indicates that it is not just doing things together, but what really has an effect is the kind of activities couples do together—how mundane versus how novel/interesting and exciting (self-expanding) it is.

## 5. Benefits of Shared Self-Expanding Activities

At the onset, romantic relationships are likely to be very self-expanding (through the novelty and excitement of the process, especially due to the inclusion of the other in the self), but typically gradually decline over months and years, creating a sense of boredom (Harasymchuk & Fehr, 2013). Relational self-expansion (as measured through the self-expansion questionnaire) is strongly related to relationship quality (Đorđević & Vlajić, 2022). However, as noted briefly, according to the self-expansion model, one way to rekindle that excitement is through shared novel and exciting activities, so that the experiences of those activities become associated with the partner and the relationship and the relationship feels deeper and closer.

This was initially tested (and supported) in a large survey (Aron et al., 2000) followed by numerous experiments and other studies. In the survey (actually conducted in the 1980s but not published until the 2000 paper which also included experiments), Aron and colleagues conducted both a newspaper survey and a door-to-door survey. In each they included a standard relationship satisfaction measure and the item “How exciting are the things you do together with your partner?” The correlations between relationship satisfaction and shared exciting activities were 0.45 and 0.51.

In the first experiment (Reissman et al., 1993), each member of the couple was given a list of activities and asked to rate each for how pleasant and how exciting it was likely to be. Couples were then randomized into a group assigned to do activities that they had both rated as exciting and pleasant or another group assigned to activities they had both rated as pleasant but not as exciting (plus a third group just serving as a control). They were then asked to do one of these (from the list assigned to them) for 1.5 h or more each week for 10 weeks. Those in the exciting activities group reported significantly greater relationship satisfaction after the 10 weeks compared to couples who did either pleasant but not exciting activities or simply the neutral control condition.

In another important early study (Aron et al., 2000), couples were invited to the lab to do an activity in one of two conditions. In one condition, they were tied together at the wrist and ankle on one side and instructed to crawl across a long mat (including over a barrier) to the other side and back 3 to 4 times; in the other condition, they completed a mundane task involving rolling a ball individually. Those doing the task together showed significantly greater relationship satisfaction (both in standard pre-post self-report measures and in researchers’ ratings of a conversation they had after—the raters were not aware of which condition they had been in).

And in another example (Coulter & Malouff, 2013), couples completed a 4-week intervention in which, each week, they engaged in a 1.5 h shared exciting activity (that the participants themselves generated) and showed greater pre- to post-intervention increases in relationship quality than couples in a wait-list control group. These are just three examples of experiments showing this basic effect.

In another example with an additional interesting finding, Muise et al. (2018), Study 3, supported the basic findings while adding some new information. Participants were randomly assigned to one of three conditions: they read information (a) about the benefits of doing self-expanding activities with their partner, (b) about doing familiar/comfortable activities with their partner, or (c) no information. Then, in the first two conditions, the participants were asked to engage in this type of activity with their partner sometime in the next 72 h. Results collected 72 h later found a substantial effect, in which the self-expanding condition predicted greater sexual desire, which strongly predicted greater relationship satisfaction (the direct effect was weak, but the mediation effect was strong).

Many other kinds of studies have also shown a strong effect on relationship satisfaction (as well as identifying various specifics). For example, one important study (Muise et al., 2018, Studies 1 and 2) used a daily diary procedure in which both members of couples each reported daily for 21 days. The study found that reported self-expansion significantly predicted higher relationship satisfaction on the same day, as well as in lagged analyses. And importantly, it did not show the reverse effect in lagged analyses, thus supporting the theoretically expected direction of the association.

In a very different kind of study, Coffey et al. (2024) examined vacations as self-expansion activities. In Study 1 they found that going on a vacation with your partner was associated with significantly greater post-vacation marital satisfaction and romantic passion than going on one without your partner. And in Study 2, they found that those who rated their trip higher on self-expansion (based on a scale concerning the amount of novel and interesting things that happened on the trip) showed significantly greater post-vacation marital satisfaction and physical intimacy.

Individual differences also matter, especially in predicting motivation to self-expand. For example, approach motivation predicts greater motivation to do self-expanding activities with one’s partner (e.g., through planning exciting dates; Harasymchuk et al., 2020; Mattingly et al., 2012). Attachment avoidance is associated with less desire for self–other overlap (Hughes et al., 2025), but is reduced over time by experiencing self-expansion in the relationships (McIntyre et al., 2025). Indeed, overall, those who plan more self-expanding activities show greater self-expansion, as has been found in daily diary studies (e.g., Harasymchuk et al., 2020) and in experiments reminding people to plan them (Harasymchuk et al., 2017).

The majority of the literature on self-expansion activities seems to operationalize them in terms of shared exciting activities (e.g., Reissman et al., 1993) or as novel activities (e.g., Muise et al., 2018). There has also been some consideration of whether challenge and arousal are necessary components of self-expansion.

Jim Graham and colleagues pointed out the importance of finding an optimal challenge level for self-expansion activities (Graham & Harf, 2015). In a series of experimental, survey, and experience sampling studies, this work shows that challenge and skill level interact to predict relationship satisfaction, such that when couples complete a moderately challenging task together that they have the skills to complete, relationship satisfaction is highest (compared both to a not very challenging task or to one they fail to be able to accomplish). Increases in positive affect after overcoming a challenge seem to be one underlying mechanism behind this relationship. This work fits with the idea that self-expansion activities can lead couples to find a flow state, which would then feed back into positive feelings about the relationship (Graham, 2008). Taken together, these findings make sense because it is satisfying to overcome a challenge and to attribute that success to one’s own (and one’s partner’s) self-efficacy while working together with a relationship partner.

Another characteristic of self-expansion activities that has been considered is the arousal level. Many of the activities that have been used to study self-expansion in the lab are physical, such as completing an obstacle course or carrying objects using chopsticks (Aron et al., 2000; Mattingly & Lewandowski, 2013). However, mental activities, such as reading novel facts or solving anagrams, have also been used to induce a sense of self-expansion in individuals (Mattingly & Lewandowski, 2013). Tomlinson et al. (2019) engaged the question of whether physiological arousal is necessary for shared self-expansion activities. In a series of four studies, they found that for both romantic couples and friend pairs, self-expansion consistently predicted relationship and individual outcomes. Although physiological arousal had some benefits, it did not appear to be necessary across studies. These results suggest that interest and/or excitement in an activity can be rewarding, but that both physical and non-physical activities could be equally beneficial (for example, Graham & Harf, 2015 found benefits for relationship satisfaction when couples engaged together in a moderately challenging puzzle task). The basic idea that novel, exciting, and interesting activities have benefits has been supported across a variety of experimental and daily diary studies; however, more work could be carried out to make recommendations on the specific types of activities that might be most beneficial for couples. Indeed, Schrage et al. (2026) found in a series of daily diary studies that for avoidantly attached individuals, novelty and excitement in the relationship attenuates typically lower relationship satisfaction. For anxiously attached individuals, in contrast, familiar and comfortable activities buffered relationship satisfaction (while exciting and novel activities did not). Thus, tailoring the type of activity to the couple’s attachment style may maximize the benefits. This kind of work could have important implications for couples in general, as well as for clinical settings, and could provide recommendations for couples’ therapy.

The majority of research on couples’ shared participation in self-expanding (novel and challenging) activities has focused on the benefits for relationship quality (e.g., Aron et al., 2000; Graham, 2008). Some work has also examined mechanisms behind the association between shared self-expansion activities and relationship satisfaction. For example, as noted earlier, Muise et al. (2018) identified sexual desire as a mediator underlying this relationship. Positive affect has also been identified as a mechanism behind the benefits of shared self-expansion (Graham & Harf, 2015; Strong & Aron, 2006). The positive affect associated with shared self-expansion activities also has implications for subjective well-being. Engaging in novel and challenging activities can be one way to see one’s partner in a new light and recapture the excitement that is typically associated with the earlier stages of relationships. There have also been some studies that have considered self-efficacy and self-expansion as outcome variables (e.g., Tomlinson et al., 2019), but more research should be carried out to consider growth as an outcome.

Relationship satisfaction and more general well-being (typically measured as subjective well-being and/or life satisfaction) seem to be tied. For example, in one recent longitudinal study using 14 years of data from the New Zealand Attitudes and Values Study, both relationship satisfaction and well-being increased leading up to marriage and then declined shortly after marriage (Dupuis et al., 2025). These results suggest that relationship and life satisfaction are linked. In addition, relationship satisfaction has also been strongly linked to physical health (Fivecoat, 2025).

One creative fMRI experiment utilized pictures of non-smoking partners as a way to help reduce activation in cigarette cue reactivity areas of the brain in smokers (Xu et al., 2012). The idea here is that the experience of self-expansion with a partner might help replace cravings due to the rewards associated with a self-expanding relationship, and indeed, former smokers report that self-expanding events played a role in quitting (Xu et al., 2010).

In another fMRI study, Xu et al. (2014) tested this using yet another creative procedure. Long-term couples were recruited in which at least one was a smoker. Before coming to the lab, the smoker was asked to abstain for at least 8 h. At the lab, the smoker was placed in the scanner and played games with their partner (who was playing their side of the game on a computer). The games were randomized to be either exciting or just pleasant (as confirmed by self-report and by brain arousal-level data). In half of each type of game there was a cigarette cue. Results showed that the cigarette cue had significantly less effect (as measured by brain response) in the exciting game condition.

There are some studies that have considered the effects of self-expansion directly on mental health and physical health. Though these studies are not focused on shared self-expanding activities, they do directly focus on self-expansion in romantic relationships. For example, McIntyre et al. (2023) found negative associations between relational self-expansion and depression across four studies that utilized a variety of sophisticated methodologies including cross-sectional, longitudinal, dyadic, and daily diary reports. Another dyadic study of Chinese newlyweds found positive actor effects linking relational self-expansion and life satisfaction one year later for both husbands and wives (Chen et al., 2025). There has also been work linking partner support for the partner’s individual self-expansion with physical health and retirement satisfaction in retired couples (Tomlinson et al., 2020). These studies imply that shared self-expansion activities would have benefits for health and well-being.

## 6. Conclusions

In this article, we focus on how doing self-expanding activities with a romantic partner can enhance relationship quality, and through that, enhance well-being. We first briefly describe the underlying self-expansion model, focusing on the motivational principle that a major basic human motive is to self-expand—increase our resources, knowledge, and so much more. We then explain that a major way we expand ourselves is through close relationships, in which the other (including their resources and such) becomes, to some extent, part of oneself (“included in the self”). However, after one develops a relationship, one gets used to this self-expansion by including the partner into the self, so the excitement of this inclusion slows over time, and thus relationships can become dull. But we argue that a major way it can be reinvigorated is by doing novel/challenging (self-expanding) activities together with our partner, so that the self-expansion becomes associated with the relationship. We then briefly review many studies (experiments, daily diary, and others) supporting the principle that shared self-expanding activities increase relationship quality. We then briefly discuss the substantial research showing that enhancing relationship quality increases well-being.

So much research has been carried out in line with the overall model, but more can be done. Some of the work on the model has been conducted in multiple cultures, but more cross-cultural research specifically with self-expansion activities would be a great addition to the literature. And which kinds of shared activities help most can also be further explored (for example, how about shared awe or shared pro-social activities). And more long-term studies could be conducted.

Particularly important in light of the focus of this paper, it will be valuable to conduct studies that specifically examine the ways in which shared self-expanding experiences lead to greater well-being for the couple members—perhaps showing a mediation effect with relationship quality as the mechanism. And ideally well-being measures might include reports by others and physiological indicators of health. And also, to promote findings in this area, in future studies on shared self-expansion in general it would be valuable to encourage authors to include measures of well-being outcomes of various kinds (where possible, both pre- and post-test measures or in diary studies at each time point) and to assess both direct and mediated patterns.

Overall, while more can be done, particularly on specific effects on well-being, the data are quite strong in showing both that relationship quality enhances well-being (including various aspects that have only been minimally studied such as self-efficacy) and the basic effect that people in romantic relationships (particularly long-term) can improve their lives by regularly doing new, novel, and interesting activities together with their partner. These findings are important both for advancing the basic understanding of relationship processes and well-being, but also for clinicians.

## Data Availability

No new data were created or analyzed in this study.
